# Associations between vaginal microecological status and high-risk human papillomavirus positivity in women from Eastern China: a retrospective cross-sectional study

**DOI:** 10.3389/fmed.2026.1890816

**Published:** 2026-07-10

**Authors:** Junxia Wang, Xinyong Liu, Fangfei Liu, Juan Liu, Yan Zhao, Cuizhu Ma, Lina Wang, Xing Shi

**Affiliations:** 1 Central Research Laboratory, The Second Qilu Hospital of Shandong University, Jinan, China; 2 Shandong Medical College, Jinan, China; 3 Zibo Maternal and Child Health Care Hospital, Zibo, China

**Keywords:** bacterial vaginosis, high-risk human papillomavirus, hrHPV genotype category, trichomonal vaginitis, vaginal microecology, vulvovaginal candidiasis

## Abstract

**Background:**

Evidence regarding the associations between vaginal microecological status and high-risk human papillomavirus (hrHPV) genotype categories remains limited, particularly in large-sample studies. Using a large hospital-based laboratory dataset with same-visit vaginal and cervical sampling, this study assessed the associations between vaginal microecological status and hrHPV infection.

**Methods:**

A total of 14,359 women were included in this retrospective cross-sectional study. Vaginal and cervical specimens collected during the same hospital visit were used for vaginal microecological assessment and hrHPV genotyping, respectively. HrHPV-positive cases were further assigned to two mutually exclusive analytical genotype categories: HPV16/18 positivity and hrHPV12 (12 non-HPV16/18 high-risk genotypes) positivity. Associations were examined using the chi-square test or Fisher’s exact test, and univariable and multivariable logistic regression analyses. Sensitivity analyses excluding women with mixed vaginitis were conducted to assess the robustness of the primary findings.

**Results:**

Overall hrHPV positivity was 15.99% (2,296/14,359, 95% CI, 15.39–16.60) and differed significantly by age (*p* < 0.001), with the highest rate in women aged ≤20 years (35.46%). Detection rates of bacterial vaginosis (BV), aerobic vaginitis (AV), trichomonal vaginitis (TV), and vulvovaginal candidiasis (VVC) also differed across age groups (all *p* < 0.001). In the multivariable analyses, BV (OR = 1.572, 95% CI: 1.052–2.218) and TV (OR = 1.738, 95% CI: 1.126–2.684) were associated with higher odds of overall hrHPV positivity after adjustment for available variables. In models stratified by hrHPV genotype category, BV (OR = 1.599, 95% CI: 1.036–2.467) and TV (OR = 1.653, 95% CI: 1.004–2.723) were associated with hrHPV12 positivity, whereas TV (OR = 1.970, 95% CI: 1.015–3.823) was associated with HPV16/18 positivity. Sensitivity analyses after excluding women with mixed vaginitis showed generally similar effect directions for the main associations.

**Conclusion:**

HrHPV positivity and vaginal microecological abnormalities showed age-specific distributions, and the associations between vaginal microecological status and hrHPV positivity showed heterogeneity across hrHPV genotype categories. These findings provide large-scale epidemiological evidence suggesting that age and hrHPV genotype category may need to be considered when interpreting the relationship between vaginal microecological status and hrHPV positivity.

## 1 Introduction

Vaginal microecology (VM) plays an important role in female reproductive health. Under healthy conditions, VM is typically dominated by *Lactobacillus* spp. By producing lactic acid, maintaining a low vaginal pH, and inhibiting the overgrowth of opportunistic pathogens, lactobacilli help form a mucosal barrier (1, 2). Disruption of this barrier may present as different types of vaginitis or vaginal dysbiosis, which may be associated with several sexually transmitted infections, including high-risk human papillomavirus (hrHPV) infection (3, 4). Persistent hrHPV infection is a key etiological factor in the development and progression of high-grade cervical precancerous lesions and cervical cancer (5). Therefore, examining the associations between vaginal microecological status and hrHPV positivity may provide epidemiological evidence for future longitudinal studies on hrHPV persistence, clearance, and cervical lesion progression.

Recent studies have suggested a close relationship between VM and hrHPV infection. *Lactobacillus*-dominated vaginal microbiota is generally associated with lower hrHPV detection rate, whereas reduced *Lactobacillus* dominance, increased anaerobic bacteria, and higher microbial diversity are more often observed in women with hrHPV infection or persistence (6–8). Longitudinal evidence has also suggested that the cervicovaginal microbiota may be associated with hrHPV clearance and the progression of hrHPV related cervical lesions (8). In China, a population-based study from Beijing (*n* = 602) reported significantly higher microbial signals of *Streptococcus*, *Prevotella*, and *Chlamydia* in HPV positive women than in healthy controls, whereas *Aerococcus* signals were lower in the HPV positive group (9). A previous hospital-based study from northwest China (*n* = 2,358) reported associations between vaginal microecological abnormalities and different HPV infection categories, including HPV16/18 and other HPV subtypes (10). However, available evidence remains limited by heterogeneity in study populations, microbiological assessments, HPV testing strategies, and confounder control. Large hospital-based laboratory cohort studies that focus on clinically relevant hrHPV genotype categories and are based on same-visit sampling remain scarce. However, previous findings may have been influenced by behavioral confounding and methodological heterogeneity, including differences in population source, sexual and reproductive factors, vaginal microbiological assessment, HPV testing methods, and adjustment for confounders.

We conducted a large retrospective cross-sectional study in Eastern China. Based on same-visit vaginal and cervical sampling and relevant clinical data, we characterized the age-specific distributions of hrHPV infection and vaginal microecological abnormalities. We further examined the associations between vaginal microecological status and hrHPV infection by genotype category.

## 2 Materials and methods

### 2.1 Study design and participant selection

This was a retrospective, hospital-based cross-sectional study conducted at the Second Hospital of Shandong University, Jinan, China. We reviewed routine clinical and laboratory records of women between January 2022 and August 2024. The study participants were women who attended the Department of Gynecology, Department of Reproductive Medicine, or Health Examination Center at our hospital. Their clinical indications were diverse and included routine gynecologic evaluation, reproductive medicine or infertility-related assessment, health screening, and evaluation of vaginitis-related symptoms. Vaginal and cervical specimens were collected during the same hospital visit and were used for vaginal microecological assessment and high-risk human papillomavirus (hrHPV) testing, respectively. Women were included if both laboratory test results and corresponding clinical information were available. If a woman had more than one eligible same-visit record during the study period, only the first record was retained for analysis.

Women were excluded if they had any of the following conditions at or before sampling: pregnancy or menstrual period; sexual intercourse, vaginal douching or local intravaginal medication within the preceding 3 days; use of antibiotics, antifungal agents, hormone replacement therapy, or oral contraceptives within the preceding 2 weeks; HIV infection or autoimmune diseases; or previous receipt of antiviral therapy, such as topical interferon therapy. These exclusion criteria were applied retrospectively based on available clinical records to reduce potential confounding in the analysis.

**Figure 1:**
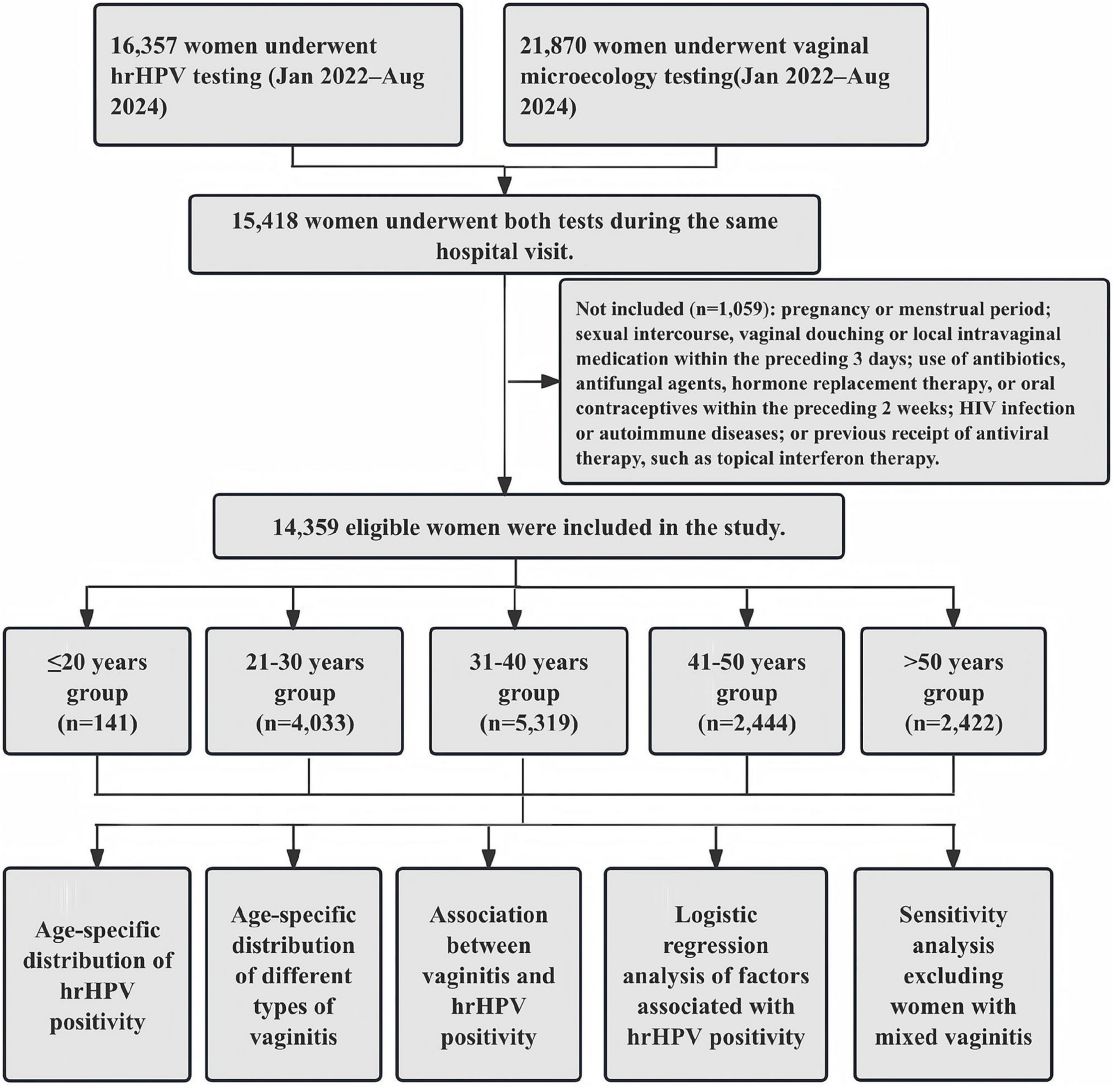
Participant selection and analysis framework. The flowchart shows the selection of participants who underwent hrHPV testing and vaginal microecological testing between January 2022 and August 2024. Women not included before determination of the final eligible cohort and the corresponding reasons are shown in the flowchart. Finally, 14,359 eligible women were included and stratified into five age groups for subsequent analyses. hrHPV, high-risk human papillomavirus.

The participant selection process is summarized in Figure 1. After identifying women who had vaginal and cervical specimens collected during the same hospital visit and applying the predefined exclusion criteria, 14,359 eligible women were included in the final analyses. The median age was 35 years, with an interquartile range (IQR) of 29–45 years. The participants were divided into five age groups: ≤20, 21–30, 31–40, 41–50, and >50 years.

This study was conducted in accordance with the Declaration of Helsinki and approved by the Scientific Research Ethics Committee of the Second Hospital of Shandong University (Approval No. KYLL-2021(KJ)P-0378). Informed consent was waived because of the retrospective design and use of de-identified data.

### 2.2 Vaginal microecological testing and diagnosis of vaginitis

Vaginal secretions were collected by qualified physicians from the upper one-third of the vagina using a vaginal swab during routine gynecologic examination. The swab was eluted in 0.5 mL of normal saline to prepare a vaginal secretion suspension. Two drops of this suspension were separately placed onto two clean glass slides for microscopic examination. One slide was prepared as a wet mount, first screened at ×100 magnification for an overall assessment and then examined at ×400 magnification for *Trichomonas vaginalis*, fungal hyphae or spores, and cellular morphology. The other slide was air-dried, fixed, Gram-stained, and examined under oil immersion (×1,000) to assess *Lactobacillus* density, background flora, microbial diversity, clue cells, Nugent score, aerobic vaginitis (AV) score, and vaginal cleanliness grade. Microscopic evaluation was performed by trained laboratory personnel in accordance with institutional standard operating procedures. In addition, biochemical indicators were assessed using a commercial vaginal microecological test kit (Shandong Stars Biological Co., Ltd., Jinan, China), including vaginal pH, leukocyte esterase (LE), hydrogen peroxide (H_2_O_2_), N-acetylglucosaminidase (NAG), and sialidase (SIA).

According to the Expert Consensus on the Diagnosis and Treatment of Mixed Vaginitis (2021 edition) and based on clinical manifestations and laboratory findings, vaginal microecological results were categorized as follows: (1) no vaginitis (NV), defined as the absence of bacterial vaginosis (BV), aerobic vaginitis (AV), trichomonal vaginitis (TV), vulvovaginal candidiasis (VVC), or any mixed vaginitis; (2) BV, defined by positive clue cells and a Nugent score ≥7; (3) AV, defined by compatible clinical manifestations and an AV score ≥3; (4) TV, defined by microscopic identification of *Trichomonas vaginalis*; (5) VVC, defined by the presence of fungal spores or hyphae on microscopy; and (6) mixed vaginitis, defined as the coexistence of two or more vaginitis types.

All microscopic examinations were performed by laboratory personnel who had received standardized training in vaginal secretion microscopy and vaginal microecological assessment. Testing was conducted according to institutional standard operating procedures, including routine internal quality control for staining, microscopy, and biochemical testing. Vaginitis classification was based on predefined laboratory and clinical diagnostic criteria. Ambiguous or discordant microscopic findings were reviewed by a senior laboratory technologist before final classification. Because hrHPV testing and vaginal microecological assessment were performed in separate laboratory workflows, laboratory staff responsible for vaginal microecological assessment were not informed of the hrHPV results at the time of interpretation. Formal inter-observer agreement statistics were not available because of the retrospective design and routine clinical laboratory setting.

### 2.3 HrHPV genotyping

Experienced clinicians exposed the cervix using a sterile vaginal speculum and gently removed excess cervical mucus with a sterile cotton swab. Exfoliated cervical cells were then collected using a cervical sampling brush, which was rotated 3–5 times during sampling. The collected cellular material was immediately transferred into a vial containing 10 mL of SurePath® preservative solution (BD Diagnostics-TriPath, Franklin Lakes, NJ, USA).

HrHPV genotyping was performed using the cobas® 4,800 HPV test (Roche Diagnostics GmbH, Mannheim, Germany) in accordance with the manufacturer’s instructions. DNA extraction, PCR amplification, and result interpretation were performed according to the standard assay protocol. The cobas® 4,800 HPV assay detects 14 high-risk HPV genotypes and separately reports HPV16 and HPV18, whereas the remaining 12 high-risk genotypes, including HPV31, 33, 35, 39, 45, 51, 52, 56, 58, 59, 66, and 68, are reported only as a pooled result. For the purposes of analysis, hrHPV infection referred to current hrHPV DNA detection at the time of sampling, defined as positivity for one or more high-risk HPV genotypes. Among hrHPV-positive participants, infections were further categorized as HPV16/18 positivity and hrHPV12 positivity, with hrHPV12 defined as the 12 non-HPV16/18 high-risk genotypes. Because HPV16/18 have higher oncogenic potential and greater clinical relevance for cervical cancer risk stratification, women with co-detection of HPV16/18 and one or more of the other 12 hrHPV genotypes were classified as HPV16/18-positive and were not double-counted in the hrHPV12-positive group.

### 2.4 Statistical analysis

Statistical analyses were performed using SPSS version 29.0. Categorical variables were compared using the chi-square test or Fisher’s exact test, as appropriate, and a two-sided *p* value <0.05 was considered statistically significant. For key prevalence estimates and subgroup proportions, 95% confidence intervals (CIs) were calculated using the exact binomial method based on the Clopper–Pearson approach. Univariable logistic regression was first performed to evaluate crude associations between demographic or vaginal microecological variables and overall hrHPV positivity, hrHPV12 positivity, and HPV16/18 positivity. Multivariable logistic regression models were then constructed using a clinically informed approach. Age group was included *a priori* in primary multivariable models because of its established relevance to both hrHPV positivity and vaginal microecological status. For laboratory and vaginal microecological variables, those with *p* < 0.05 in univariable analyses were entered into the primary multivariable logistic regression models. Statistical significance in multivariable analyses was defined as a two-sided *p* value <0.05, and odds ratios (ORs) with 95% confidence intervals (CIs) were calculated. Sensitivity analyses were performed after excluding women with mixed vaginitis to assess the robustness of the primary findings. Figures were generated using GraphPad Prism and Python.

## 3 Results

### 3.1 Age distribution of hrHPV infection

**Figure 2:**
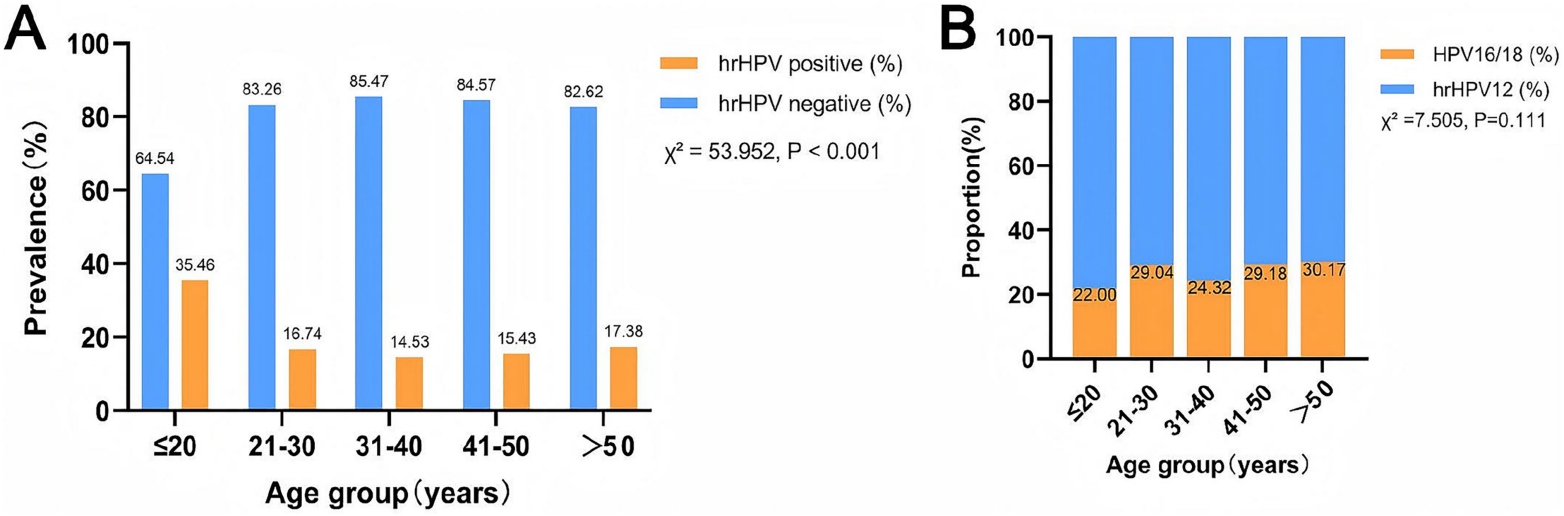
Age-specific distributions of hrHPV infection and hrHPV genotype categories, **(A)** Age-specific distribution of overall hrHPV infection. **(B)** Age-stratified distribution of HPV16/18 and hrHPV12 among hrHPV-positive women. Differences across age groups were assessed using the chi-square test. hrHPV, high-risk human papillomavirus; hrHPV12, 12 non-HPV16/18 high-risk HPV genotypes. Exact binomial 95% CIs for age-specific proportions are provided in Supplementary Tables S1, S2.

Among 14,359 women, the prevalence of hrHPV infection was 15.99% (2,296/14,359, 95% CI, 15.39–16.60) and differed significantly across age groups (*p* < 0.001). The highest prevalence was observed in women aged ≤20 years (35.46%, 50/141, 95% CI, 27.59–43.95), whereas the lowest prevalence was observed in the 31-40-year group (14.53%, 773/5,319, 95% CI, 13.60–15.51). A modest increase was observed in women aged >50 years, with a prevalence of 17.38% (421/2,422, 95% CI, 15.89–18.95) (Figure 2A; Supplementary Table S1). Women aged ≤20 years had a relatively small sample size relative to other age groups, potentially leading to low precision of statistical estimates. The overall HPV16/18 and hrHPV12 infection rates were 4.40% (632/14,359) and 11.59% (1,664/14,359), respectively. Among hrHPV-positive women, HPV16/18 accounted for 27.53% (632/2,296), including 204 women with co-detection of HPV16/18 and one or more of the other 12 high-risk genotypes. The infection rates of HPV16/18 and hrHPV12 did not vary significantly across age groups. Across all age groups, hrHPV12 positivity was approximately 2.3–3.5 times that of HPV16/18 positivity (Figure 2B; Supplementary Table S2).

### 3.2 Age distribution of different types of vaginitis

**Figure 3:**
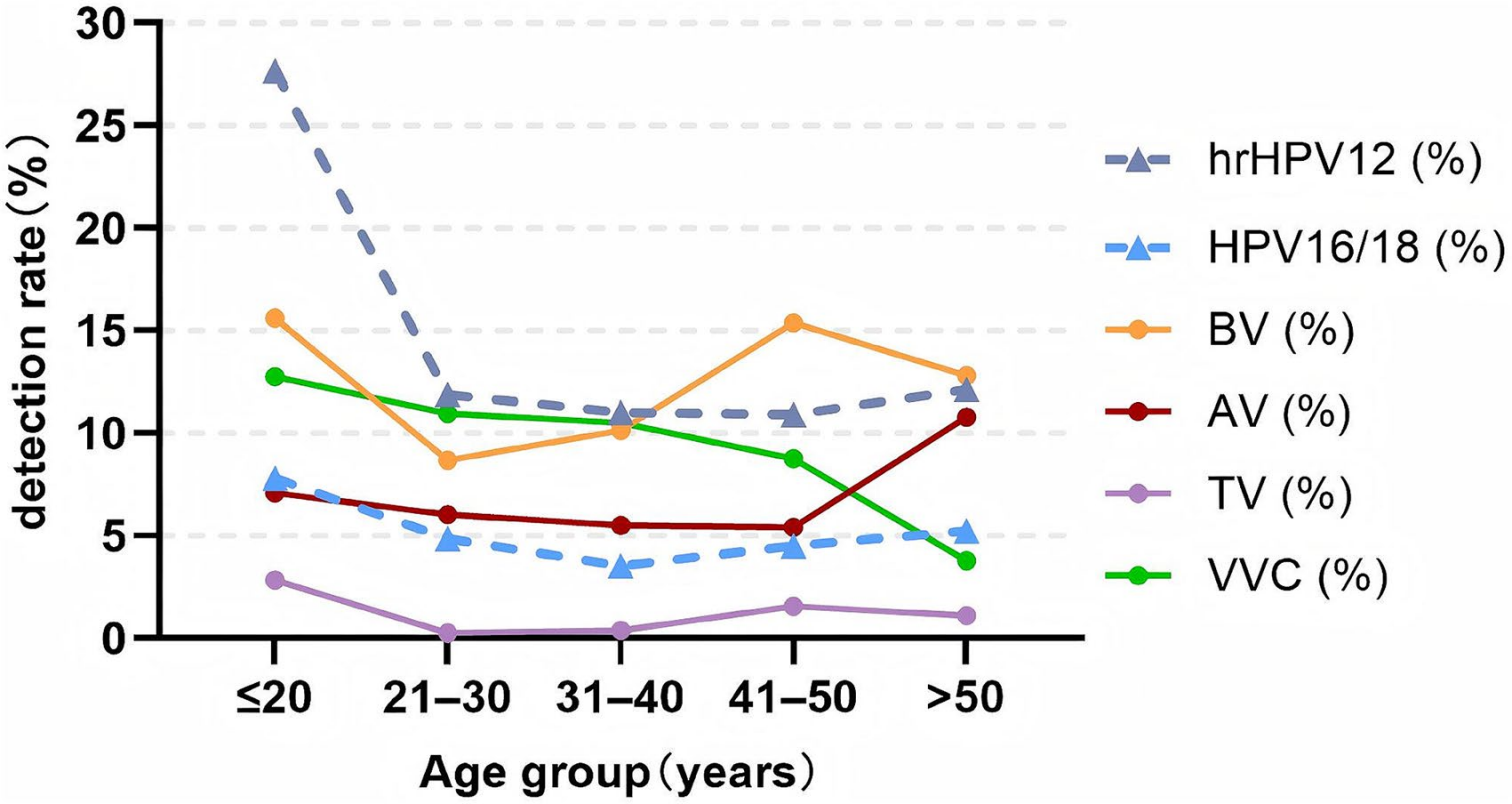
Age-specific detection rates of vaginitis subtypes and positivity rates of hrHPV genotype categories. Line plot showing the detection rates of BV, AV, TV, and VVC, as well as the positivity rates of HPV16/18 and hrHPV12, across five age groups. Differences across age groups were assessed using the chi-square test. hrHPV12, 12 non-HPV16/18 high-risk HPV genotypes; BV, bacterial vaginosis; AV, aerobic vaginitis; TV, trichomonal vaginitis; VVC, vulvovaginal candidiasis. Exact binomial 95% CIs for age-specific proportions are provided in Supplementary Tables S1–S3.

The detection rates of BV, AV, TV, and VVC differed significantly across age groups (all *p* < 0.001). BV was detected in 11.13% of women overall (1,598/14,359, 95% CI, 10.62–11.65), with the highest rate observed in women aged ≤20 years (15.60%). AV accounted for 6.55% of cases (941/14,359, 95% CI, 6.15–6.97) and was most frequent in women aged >50 years (10.78%). TV was uncommon overall, with a detection rate of 0.70% (100/14,359, 95% CI, 0.57–0.85), but its highest rate was observed in women aged ≤20 years (2.84%). Because both the number of TV-positive cases and the size of the ≤20-year age group were small, the higher TV positivity observed in this age group may reflect either a true trend or random variation due to sparse data. Therefore, the numbers of women with TV alone and mixed vaginitis involving TV, including BV + TV (*n* = 39), AV + TV (*n* = 12), and BV + AV + TV (*n* = 16), were relatively small. Estimates for these sparse categories may be unstable, and statistically significant findings may reflect limited precision rather than robust associations. VVC was detected in 9.23% of participants (1,325/14,359, 95% CI, 8.76–9.71) and declined with increasing age, from 12.77% in women aged ≤20 years to 3.80% in those aged >50 years (Figure 3; Supplementary Table S3).

### 3.3 Differences in hrHPV positivity according to vaginal microecological status and hrHPV genotype category

**Figure 4:**
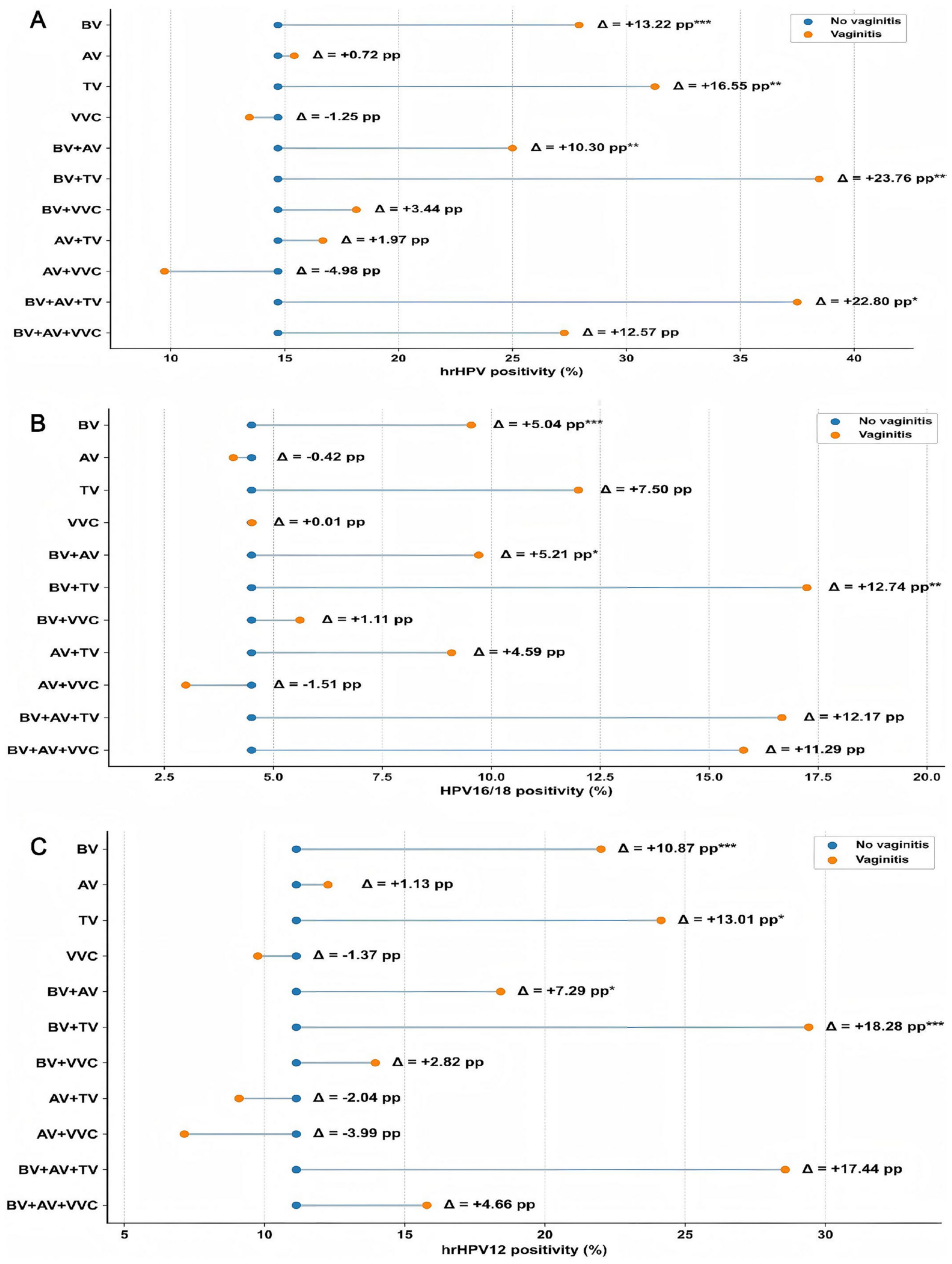
HrHPV positivity according to vaginal microecological status. Dumbbell plots showing differences in **(A)** overall hrHPV positivity, **(B)** HPV16/18 positivity, and **(C)** hrHPV12 positivity between the no vaginitis group and different vaginitis subgroups. *Δ* values indicate absolute percentage-point differences compared with the no vaginitis group. *p* values were calculated using the chi-square test or Fisher’s exact test. hrHPV, high-risk human papillomavirus; hrHPV12, 12 non-HPV16/18 high-risk HPV genotypes; NV, no vaginitis; BV, bacterial vaginosis; AV, aerobic vaginitis; TV, trichomonal vaginitis; VVC, vulvovaginal candidiasis; pp., percentage points. *p < 0.05, **p < 0.01, ***p < 0.001. The NV group was used as the reference category. Estimates for subgroups with small counts, particularly BV + TV, AV + TV, and BV + AV + TV, and AV + TV + VVC, should be interpreted cautiously because of limited statistical precision. Exact binomial 95% CIs for subgroup-specific proportions are provided in Supplementary Tables S4–S6.

In the NV group, overall hrHPV positivity was 14.70% (1,609/10,946). Compared with the NV group, higher overall hrHPV detection was observed in the BV, TV, BV + AV, BV + TV, and BV + AV + TV groups. The largest difference was found in the BV + TV group (38.46%, *Δ* = +23.76 pp, *p* < 0.001), while a notable increase was also observed in the BV group (27.92%, Δ = +13.22 pp, *p* < 0.001). Overall hrHPV positivity was lower in the VVC and AV + VVC groups than in the NV group, but these differences were not statistically significant (Figure 4A; Supplementary Table S4).

For outcomes by hrHPV genotype category, HPV16/18 positivity in the NV group was 4.50% (440/9,777), and was significantly higher in the BV, BV + AV, and BV + TV groups than in the NV group (*p* < 0.05). BV + TV group (17.24%, *Δ* = +12.74 pp, *p* = 0.009) showed the largest difference (Figure 4B; Supplementary Table S5). Compared with the NV group (11.13%, 1,169/10,506), hrHPV12 positivity was significantly higher in the BV, TV, BV + AV, and BV + TV groups (*p* < 0.05). The largest difference was observed in BV + TV (29.41%, Δ = +18.28 pp, *p* < 0.001) (Figure 4C; Supplementary Table S6). Non-significant subgroup comparisons are presented in Supplementary Tables S4–S6.

Some mixed-infection subgroups were small, such as BV + TV (*n* = 39), AV + TV (*n* = 12), BV + AV + TV (*n* = 16), which limited the precision of subgroup comparisons. Therefore, these results should be interpreted cautiously. The AV + TV + VVC subgroup was not shown in the dumbbell plot because it included only one case, but the corresponding data are provided in Supplementary Tables S4–S6.

### 3.4 Differences in hrHPV infection between single-type and mixed vaginitis

Among women with single-type vaginitis, hrHPV infection rate was 19.96% (579/2,901) and differed significantly across vaginitis subtypes (*p* < 0.001). Higher infection rates were observed in TV (31.25%, 10/32) and BV (27.92%, 327/1,171), whereas lower rates were found in AV (15.42%, 107/694) and VVC (13.45%, 135/1,004). Among women with mixed vaginitis, hrHPV infection rate was 21.09% (108/512) and also differed across subtypes (*p* = 0.006). The highest rates were observed in BV + TV (38.46%, 15/39) and BV + AV + TV (37.50%, 6/16), whereas AV + VVC showed the lowest rate (9.72%, 7/72). The AV + TV + VVC subgroup included only one participant and showed no hrHPV positivity (Table 1). The proportion of HPV16/18 among hrHPV-positive women did not differ significantly across vaginitis subtypes in either the single-type or mixed-type vaginitis groups (Table 2).

**Table 1:** HrHPV infection in women with single-type and mixed vaginitis subtypes.

Vaginitis	Total *n*	hrHPV		χ^2^	*p*-value
		Negative (%)	Positive (%; 95% CI)		
Mixed vaginitis	512	404 (78.91)	108 (21.09; 17.64–24.89)	18.608	**0.006**
BV + AV	124	93 (75.00)	31 (25.00; 17.66–33.57)		
BV + TV	39	24 (61.54)	15 (38.46; 23.36–55.38)		
BV + VVC	226	185 (81.86)	41 (18.14; 13.34–23.80)		
AV + TV	12	10 (83.33)	2 (16.67; 2.09–48.41)		
AV + VVC	72	65 (90.28)	7 (9.72; 4.00–19.01)		
BV + AV + TV	16	10 (62.50)	6 (37.50; 15.20–64.57)		
BV + AV + VVC	22	16 (72.73)	6 (27.27; 10.73–50.22)		
AV + TV + VVC	1	1 (100.00)	0 (0.00; 0.00–97.50)		
Single vaginitis	2,901	2,322 (80.04)	579 (19.96; 18.52–21.46)	84.683	**<0.001**
BV	1,171	844 (72.08)	327 (27.92; 25.37–30.59)		
AV	694	587 (84.58)	107 (15.42; 12.81–18.32)		
TV	32	22 (68.75)	10 (31.25; 16.12–50.01)		
VVC	1,004	869 (86.55)	135 (13.45; 11.40–15.71)		

hrHPV, high-risk human papillomavirus; BV, bacterial vaginosis; AV, aerobic vaginitis; TV, trichomonal vaginitis; VVC, vulvovaginal candidiasis. 95% CIs for proportions were calculated using the exact binomial (Clopper–Pearson) method. Estimates for subgroups with small counts, particularly BV + TV, AV + TV, and BV + AV + TV, and AV + TV + VVC, should be interpreted cautiously because of limited statistical precision. Bold values indicate *p* < 0.05.

**Table 2:** Distribution of HPV16/18 and hrHPV12 among hrHPV-positive women with single-type and mixed vaginitis subtypes.

Vaginitis	Total *n*	hrHPV positive		χ^2^	*p*-value
		hrHPV12 (%; 95% CI)	HPV16/18 (%; 95% CI)		
Mixed vaginitis	108	74 (68.52; 58.88–77.12)	34 (31.48; 22.88–41.12)	2.474	0.908
BV + AV	31	21 (67.74; 48.63–83.32)	10 (32.26; 16.68–51.37)		
BV + TV	15	10 (66.67; 38.38–88.18)	5 (33.33; 11.82–61.62)		
BV + VVC	41	30 (73.17; 57.06–85.78)	11 (26.83; 14.22–42.94)		
AV + TV	2	1 (50.00; 1.26–98.74)	1 (50.00; 1.26–98.74)		
AV + VVC	7	5 (71.43; 29.04–96.33)	2 (28.57; 3.67–70.96)		
BV + AV + TV	6	4 (66.67; 22.28–95.67)	2 (33.33; 4.33–77.72)		
BV + AV + VVC	6	3 (50.00; 11.81–88.19)	3 (50.00; 11.81–88.19)		
Single vaginitis	579	421 (72.71; 68.89–76.30)	158 (27.29; 23.70–31.11)	1.618	0.657
BV	327	238 (72.78; 67.61–77.53)	89 (27.22; 22.47–32.39)		
AV	107	82 (76.64; 67.47–84.27)	25 (23.36; 15.73–32.53)		
TV	10	7 (70.00; 34.75–93.33)	3 (30.00; 6.67–65.25)		
VVC	135	94 (69.63; 61.13–77.24)	41 (30.37; 22.76–38.87)		

hrHPV, high-risk human papillomavirus; hrHPV12, 12 non-HPV16/18 high-risk HPV genotypes; BV, bacterial vaginosis; AV, aerobic vaginitis; TV, trichomonal vaginitis; VVC, vulvovaginal candidiasis. 95% CIs for proportions were calculated using the exact binomial (Clopper–Pearson) method. Estimates for subgroups with small counts, particularly BV + TV, AV + TV, and BV + AV + TV, should be interpreted cautiously because of limited statistical precision. Bold values indicate *p* < 0.05.

### 3.5 Factors associated with hrHPV infection

#### 3.5.1 Univariable analysis

In univariable analyses, SIA positivity, LE positivity, BV, and TV were associated with higher odds of overall hrHPV infection (*p* < 0.001), whereas VVC showed an inverse association trend (*p* = 0.072; Table 3). Similar patterns were observed for hrHPV12. SIA positivity, LE positivity, BV, and TV were associated with increased odds of hrHPV12 infection (all *p* < 0.001), VVC again showed a borderline inverse association (*p* = 0.05; Table 4). For HPV16/18, abnormal vaginal pH, SIA positivity, LE positivity, BV, and TV were associated with HPV16/18 positivity (*p* < 0.05; Table 5). Age-specific differences in overall hrHPV (all *p* < 0.001), hrHPV12 (all *p* < 0.001), and HPV16/18 (all *p* < 0.1) positivity were observed across Tables 3–5, with women aged ≤20 years consistently showing higher positivity rates than the older age groups.

**Table 3:** Factors associated with overall hrHPV infection in univariable and multivariable logistic regression analyses.

Variables	Total *n*	hrHPV		Univariate analysis		Multivariate analysis	
		Positive (%)	Negative (%)	*p*-value	OR (95% CI)	*p*-value	OR (95% CI)
NAG	N: 12,939	2,072 (16.01)	10,867 (83.99)	0.816	0.982 (0.845–1.142)		
	A: 1,420	224 (15.77)	1,196 (84.23)				
PH	N: 4,394	706 (16.07)	3,688 (83.93)	0.867	0.992 (0.900–1.092)		
	A: 9,965	1,590 (15.96)	8,375 (84.04)				
Age groups (years)	≤20: 141	50 (35.46)	91 (64.54)				
	21–30: 4,033	675 (16.74)	3,358 (83.26)	**<0.001**	0.366 (0.257–0.522)	**<0.001**	0.399 (0.278–0.571)
	31–40: 5,319	773 (14.53)	4,546 (85.47)	**<0.001**	0.309 (0.217–0.441)	**<0.001**	0.332 (0.232–0.476)
	41–50: 2,444	377 (15.43)	2,067 (84.57)	**<0.001**	0.332 (0.231–0.477)	**<0.001**	0.339 (0.235–0.490)
	>50: 2,422	421 (17.38)	2,001 (82.62)	**<0.001**	0.383 (0.267–0.549)	**<0.001**	0.397 (0.275–0.572)
*Lactobacilli*	None: 11,950	1915 (16.03)	10,035 (83.97)				
	1+: 1,246	193 (15.49)	1,053 (84.51)	0.623	0.960 (0.818–1.128)		
	2+: 975	154 (15.79)	821 (84.21)	0.85	0.983 (0.822–1.176)		
	3+: 188	34 (18.09)	154 (81.91)	0.446	1.157 (0.795–1.683)		
H_2_O_2_	N: 729	114 (15.64)	615 (84.36)	0.79	1.028 (0.838–1.262)		
	A: 13,630	2,182 (16.01)	11,448 (83.99)				
SIA	N: 12,605	1,839 (14.59)	10,766 (85.41)	**<0.001**	2.063 (1.834–2.320)	0.106	1.348 (0.938–1.938)
	A: 1,754	457 (26.05)	1,297 (73.95)				
LE	N: 4,394	601 (13.68)	3,793 (86.32)	**<0.001**	1.294 (1.170–1.431)	**<0.001**	1.211 (1.094–1.341)
	A: 9,965	1,695 (17.01)	8,270 (82.99)				
BV	N: 12,761	1,870 (14.65)	10,891 (85.35)	**<0.001**	2.117 (1.875–2.390)	**0.026**	1.572 (1.052–2.218)
	A: 1,598	426 (26.66)	1,172 (73.34)				
AV	N: 13,418	2,137 (15.93)	11,281 (84.07)	0.432	1.073 (0.900–1.281)		
	A: 941	159 (16.90)	782 (83.10)				
TV	N: 14,259	2,263 (15.87)	11,996 (84.13)	**<0.001**	2.611 (1.717–3.971)	**0.013**	1.738 (1.126–2.684)
	A: 100	33 (33.00)	67 (67.00)				
VVC	N: 13,034	2,107 (16.17)	10,927 (83.83)	0.072	0.863 (0.735–1.013)		
	A: 1,325	189 (14.26)	1,136 (85.74)				

hrHPV, high-risk human papillomavirus; BV, bacterial vaginosis; AV, aerobic vaginitis; TV, trichomonal vaginitis; VVC, vulvovaginal candidiasis; NAG, N-acetylglucosaminidase; SIA, sialidase; LE, leukocyte esterase; N, normal; A, abnormal; CI, confidence interval; OR, odds ratio. Age group was included a priori in multivariable model. Variables with *p* < 0.05 in univariate logistic regression were entered into the multivariable logistic regression model. For age group, ≤20 years was used as the reference category. For *Lactobacilli*, the “None” group was used as the reference category. For binary variables, the negative or normal group was used as the reference category. Bold values: *p* < 0.05.

**Table 4:** Factors associated with hrHPV12 infection in univariable and multivariable logistic regression analyses.

Variables	Total *n*	hrHPV12 positive (%)	hrHPV negative (%)	Univariate analysis		Multivariate analysis	
				*p*-value	OR (95% CI)	*p*-value	OR (95% CI)
NAG	N: 12,373	1,506 (12.17)	10,867 (87.83)	0.591	0.953 (0.801–1.135)		
	A: 1,354	158 (11.67)	1,196 (88.33)				
PH	N: 4,225	537 (12.71)	3,688 (87.29)	0.159	0.924 (0.828–1.031)		
	A: 9,502	1,127 (11.86)	8,375 (88.14)				
Age groups (years)	≤20: 130	39 (30.00)	91 (70.00)				
	21–30: 3,837	479 (12.48)	3,358 (87.52)	**<0.001**	0.333 (0.226–0.490)	**<0.001**	0.359 (0.242–0.530)
	31–40: 5,131	585 (11.40)	4,546 (88.60)	**<0.001**	0.300 (0.204–0.441)	**<0.001**	0.318 (0.215–0.469)
	41–50: 2,334	267 (11.44)	2,067 (88.56)	**<0.001**	0.301 (0.203–0.448)	**<0.001**	0.305 (0.204–0.455)
	>50: 2,295	294 (12.81)	2,001 (87.19)	**<0.001**	0.343 (0.231–0.509)	**<0.001**	0.350 (0.235–0.522)
*Lactobacilli*	None: 11,416	1,381 (12.10)	10,035 (87.90)				
	1+: 1,193	140 (11.74)	1,053 (88.26)	0.715	0.966 (0.803–1.163)		
	2+: 940	119 (12.66)	821 (87.34)	0.612	1.053 (0.862–1.287)		
	3+: 178	24 (13.48)	154 (86.52)	0.574	1.132 (0.734–1.747)		
H_2_O_2_	N: 703	88 (12.52)	615 (87.48)	0.741	0.962 (0.765–1.210)		
	A: 13,024	1,576 (12.10)	11,448 (87.90)				
SIA	N: 12,103	1,337 (11.05)	10,766 (88.95)	**<0.001**	2.030 (1.776–2.321)	0.249	1.282 (0.841–1.956)
	A: 1,624	327 (20.14)	1,297 (79.86)				
LE	N: 4,238	445 (10.50)	3,793 (89.50)	**<0.001**	1.256 (1.120–1.410)	**0.006**	1.178 (1.049–1.324)
	A: 9,489	1,219 (12.85)	8,270 (87.15)				
BV	N: 12,249	1,358 (11.09)	10,891 (88.91)	**<0.001**	2.094 (1.824–2.404)	**0.034**	1.599 (1.036–2.467)
	A: 1,478	306 (20.70)	1,172 (79.30)				
AV	N: 12,829	1,548 (12.07)	11,281 (87.93)	0.45	1.081 (0.883–1.323)		
	A: 898	116 (12.92)	782 (87.08)				
TV	N: 13,638	1,642 (12.04)	11,996 (87.96)	**<0.001**	2.399 (1.478–3.894)	0.048	1.653 (1.004–2.723)
	A: 89	22 (24.72)	67 (75.28)				
VVC	N: 12,459	1,532 (12.30)	10,927 (87.70)	0.05	0.829 (0.687–1.000)		
	A: 1,268	132 (10.41)	1,136 (89.59)				

hrHPV, high-risk human papillomavirus; hrHPV12, 12 non-HPV16/18 high-risk HPV genotypes; BV, bacterial vaginosis; AV, aerobic vaginitis; TV, trichomonal vaginitis; VVC, vulvovaginal candidiasis; NAG, N-acetylglucosaminidase; SIA, sialidase; LE, leukocyte esterase; N, normal; A, abnormal; CI, confidence interval; OR, odds ratio. Age group was included a priori in multivariable model. Variables with *p* < 0.05 in univariate logistic regression were entered into the multivariable logistic regression model. For age group, ≤20 years was used as the reference category. For *Lactobacilli*, the “None” group was used as the reference category. For binary variables, the negative or normal group was used as the reference category. Bold values: *p* < 0.05.

**Table 5:** Factors associated with HPV16/18 infection in univariable and multivariable logistic regression analyses.

Variables	Total *n*	HPV16/18 positive (%)	hrHPV negative (%)	Univariate analysis		Multivariate analysis	
				*p*-value	OR (95% CI)	*p*-value	OR (95% CI)
NAG	N: 11,433	566 (4.95)	10,867 (95.05)	0.665	1.06 (0.815–1.377)		
	A: 1,262	66 (5.23)	1,196 (94.77)				
PH	N: 3,857	169 (4.38)	3,688 (95.62)	**0.041**	1.206 (1.007–1.445)	0.615	1.049 (0.870–1.266)
	A: 8,838	463 (5.24)	8,375 (94.76)				
Age groups (years)	≤20: 102	11 (10.78)	91 (89.22)				
	21–30: 3,554	196 (5.51)	3,358 (94.49)	**0.026**	0.483 (0.254–0.918)	0.061	0.537 (0.280–1.029)
	31–40: 4,734	188 (3.97)	4,546 (96.03)	**0.001**	0.342 (0.180–0.650)	**0.003**	0.376 (0.196–0.720)
	41–50: 2,177	110 (5.05)	2,067 (94.95)	**0.014**	0.440 (0.229–0.847)	**0.021**	0.458 (0.236–0.887)
	>50: 2,128	127 (5.97)	2,001 (94.03)	0.052	0.525 (0.274–1.007)	0.072	0.546 (0.282–1.056)
*Lactobacilli*	None: 10,569	534 (5.05)	10,035 (94.95)				
	1+: 1,106	53 (4.79)	1,053 (95.21)	0.706	0.946 (0.708–1.263)		
	2+: 856	35 (4.09)	821 (95.91)	0.213	0.801 (0.565–1.136)		
	3+: 164	10 (6.10)	154 (93.90)	0.546	1.220 (0.640–2.327)		
H_2_O_2_	N: 641	26 (4.06)	615 (95.94)	0.272	1.252 (0.839–1.870)		
	A: 12,054	606 (5.03)	11,448 (94.97)				
SIA	N: 11,268	502 (4.46)	10,766 (95.54)	**<0.001**	2.150 (1.758–2.629)	0.184	1.510 (0.822–2.775)
	A: 1,427	130 (9.11)	1,297 (90.89)				
LE	N: 3,949	156 (3.95)	3,793 (96.05)	**<0.001**	1.399 (1.163–1.684)	**0.006**	1.299 (1.077–1.566)
	A: 8,746	476 (5.44)	8,270 (94.56)				
BV	N: 11,403	512 (4.49)	10,891 (95.51)	**<0.001**	2.178 (1.769–2.681)	0.337	1.360 (0.726–2.545)
	A: 1,292	120 (9.29)	1,172 (90.71)				
AV	N: 11,870	589 (4.96)	11,281 (95.04)	0.75	1.053 (0.766–1.447)		
	A: 825	43 (5.21)	782 (94.79)				
TV	N: 12,617	621 (4.92)	11,996 (95.08)	**<0.001**	3.171 (1.668–6.031)	**0.045**	1.970 (1.015–3.823)
	A: 78	11 (14.10)	67 (85.90)				
VVC	N: 11,502	575 (5.00)	10,927 (95.00)	0.738	0.954 (0.721–1.260)		
	A: 1,193	57 (4.78)	1,136 (95.22)				

hrHPV, high-risk human papillomavirus; BV, bacterial vaginosis; AV, aerobic vaginitis; TV, trichomonal vaginitis; VVC, vulvovaginal candidiasis; NAG, N-acetylglucosaminidase; SIA, Sialidase; LE, leukocyte esterase; N, normal; A, abnormal; CI, confidence interval; OR, odds ratio. Age group was included a priori in multivariable model. Variables with *p* < 0.05 in univariate logistic regression were entered into the multivariable logistic regression model. For age group, ≤20 years was used as the reference category. For *Lactobacilli*, the “None” group was used as the reference category. For binary variables, the negative or normal group was used as the reference category. Bold values: *p* < 0.05.

#### 3.5.2 Multivariable analysis

In the model for overall hrHPV positivity, LE positivity (OR = 1.211, 95% CI: 1.094–1.341, *p* < 0.001), BV (OR = 1.572, 95% CI: 1.052–2.218, *p* = 0.026) and TV (OR = 1.738, 95% CI: 1.126–2.684, *p* = 0.013) were associated with overall hrHPV infection. Age-specific differences remained evident after adjustment, with women aged ≤20 years showing higher odds of overall hrHPV infection than the older age groups (all *p* < 0.001; Table 3). The statistically significant positive findings in Table 4 showed that LE positivity (OR = 1.178, 95% CI: 1.049–1.324, *p* = 0.006), BV (OR = 1.599, 95% CI: 1.036–2.467, *p* = 0.034) and TV (OR = 1.653, 95% CI: 1.004–2.723, *p* = 0.048) were associated with higher odds of hrHPV12 infection after multivariable adjustment. Age-related differences were generally consistent with those observed for overall hrHPV infection (all *p* < 0.001). Table 5 showed that LE positivity (OR = 1.299, 95% CI: 1.077–1.566, p = 0.006) remained associated with HPV16/18 infection in the multivariable model. A similar positive association was observed for TV (OR = 1.970, 95% CI: 1.015–3.823, *p* = 0.045). Complete adjusted ORs, 95% CIs, and *p* values for all variables included in the multivariable models are presented in Tables 3–5. After women with mixed vaginitis were excluded, sensitivity analyses showed generally similar directions for the main associations, particularly the age-specific patterns and the associations of LE positivity, BV, and TV with overall hrHPV infection. However, some genotype-specific findings, especially those involving TV and VVC, may be affected by sparse-category estimates because of small case numbers (Supplementary Tables S7–S9).

## 4 Discussion

Persistent infection with hrHPV is the main cause of cervical cancer. HPV16/18 accounts for a large proportion of invasive cervical cancer cases worldwide (11). In this context, age-specific patterns of hrHPV infection, especially HPV16/18 infection, are relevant to cervical cancer prevention and screening. In our study, the prevalence of hrHPV infection differed significantly across age groups. The highest positivity rate was observed in women aged ≤20 years, followed by lower rates in the middle age groups and a modest increase in women aged >50 years. The observed age distribution is generally consistent with previous epidemiological studies (12–14). We found that the prevalence of hrHPV infection in women aged ≤20 years was approximately 2.0–2.4 times that in the other age groups. This may partly reflect greater HPV exposure in younger women, including earlier sexual debut and behavioral factors related to HPV acquisition (15). This finding is also relevant to the WHO global cervical cancer elimination strategy. One of the strategy targets is to achieve 90% HPV vaccination coverage among girls by 15 years of age by 2030 (16). The modest increase in women aged >50 years may be related to immunosenescence and menopause-related changes in the vaginal microenvironment (17). These changes may be related to local mucosal defense and hrHPV detection or clearance, thereby providing a possible biological context for the age-related heterogeneity in hrHPV infection observed in this study. We also found that, among women with hrHPV infection, HPV16/18 accounted for the highest proportion in the >50-year age group (30.17%). Chinese cancer registry data have shown that cervical cancer incidence peaks at 50–54 years, which may provide some contextual support for this finding (18). Given the higher oncogenic potential of HPV16/18, this pattern may be relevant to cervical cancer risk stratification among middle-aged and older women in China. Meanwhile, our findings also indicate that cervical cancer screening should not be overlooked among older women, particularly HPV-based testing.

When placed in an international context, our findings show both similarities and differences compared with reports from other regions. A recent study from Greece reported an overall HR-HPV positivity rate of 8.8% among 3,500 women aged 30–50 years, with the highest rate observed in the youngest age group included in that study (31–35 years, 25.5%) (19). In Brazil, an organized screening program involving 20,398 unvaccinated women aged 25–64 years reported an overall hrHPV prevalence of 12.8% and a bimodal age pattern, with a first peak at 25–26 years and a smaller second peak at 55–56 years (20). These findings are partly consistent with our observation. However, the overall hrHPV positivity in our hospital-based clinical laboratory population from Eastern China (15.99%) was higher than that reported in the Greek and Brazilian studies. Studies from African populations have shown different epidemiological patterns. A hospital-based study from Northcentral Ethiopia reported an HPV16/18 positivity rate of 13.4%, which was higher than the HPV16/18 detection rate observed in our cohort (4.40%) (21). However, direct comparison between the two studies is limited because that study tested only for HPV16/18 and did not include all 14 high-risk HPV genotypes. These differences may reflect geographic variation in sexual behavior, HPV vaccination coverage, HIV and other sexually transmitted infection burden, population age structure, and HPV testing methods. Therefore, although the age-related pattern observed in our study is broadly consistent with international evidence, absolute prevalence estimates should be compared cautiously across regions and study designs.

We observed age-related differences in the detection rates of BV, AV, TV, and VVC. AV was most common in women aged >50 years. BV, VVC, and TV were most frequently detected in women aged ≤20 years and TV was uncommon overall. A recent Canadian study reported partly consistent findings (22). The relatively high frequency of BV in women aged ≤20 years may partly reflect age-related differences in sexual exposure and genital hygiene practices. Previous studies have reported associations between BV and younger age at first intercourse, new or multiple sexual partners, unprotected sex, and vaginal douching (23, 24). VVC is viewed as an opportunistic overgrowth of *Candida albicans* (25). Experimental studies have shown that estrogen can enhance *C. albicans* morphogenesis and vaginal colonization (26, 27). For AV, the higher detection rate in older women is consistent with the need to consider menopause-related vaginal microecological changes in clinical assessment. A recent cross-sectional study reported a markedly higher prevalence of AV in postmenopausal than in premenopausal women (28). These observations correspond to the age-related trends of VVC and AV observed in our study.

We found that TV was detected in 0.70% of women overall, with the highest proportion observed in women aged ≤20 years (2.84%). Because the number of TV positive cases in our study was small, this finding should be further validated in larger studies. In population-based settings, TV is usually uncommon. U. S. population-based data showed that the prevalence of *T. vaginalis* infection was 1.8% among women (29). In the British study, the weighted prevalence of TV was 0.3% among women aged 16–44 years, with the highest rate observed in those aged 16–24 years (0.6%) (30). The higher rate in younger women may reflect age-related differences in sexual exposure and transmission risk. Our findings indicate that age-related differences were not limited to hrHPV positivity. Similar patterns were also observed in these vaginal microecological abnormalities. These age-related patterns may support the relevance of sexual health and genital hygiene education for young women.

In the multivariable models, BV and TV remained related to overall hrHPV-positive status after adjustment for available variables. The associations were not completely uniform between HPV16/18 and hrHPV12. BV and TV were more evident for hrHPV12 positivity, whereas TV was related to HPV16/18-positive status. However, the genotype-specific associations involving TV were less stable in sensitivity analyses, which may be partly explained by the small number of TV-positive cases. Therefore, the observed association between TV and hrHPV genotype categories requires validation in larger studies. BV is characterized by reduced Lactobacillus dominance and enrichment of anaerobic or dysbiosis-associated bacteria (31). These changes may alter vaginal pH, impair mucosal barrier function, and modify local inflammatory or immune responses (32, 33), thereby providing a possible biological context for the positive association between BV and hrHPV positivity observed in our study. The positive relationship between BV and hrHPV detection is supported by previous evidence. A systematic review and meta-analysis reported a positive association between BV and HPV infection (4). More recent evidence focusing on hrHPV infection also reported a significant association for BV, whereas the results for TV and VVC were not statistically significant (34). This discrepancy from our TV finding may be related to differences in study population, outcome definition, laboratory methods, statistical adjustment, or the limited number of TV positive cases. Therefore, the observed associations between TV and hrHPV, particularly HPV16/18, require further validation in larger studies. Experimental studies suggest that *T. vaginalis* may disrupt epithelial junctions and impair mucosal barrier integrity (35), potentially enhancing viral access to the cervical epithelium (36). These findings may provide a possible biological context for the associations observed in the present study.

VVC showed a negative trend in association with hrHPV infection in both the multivariable logistic regression and sensitivity analyses, although these associations did not reach statistical significance. The VVC group also had a lower overall hrHPV positivity rate than the NV group. Evidence on VVC and HPV infection remains inconsistent. Some studies have reported no clear relationship between VVC and HPV infection (34, 37). Recent evidence suggests that *C. albicans* may show different patterns in relation to concurrent HPV detection and HPV persistence (38). Gao et al. found that vaginal Candida infection was negatively associated with HPV infection after adjustment (OR = 0.62, 95% CI: 0.48–0.80), which is broadly consistent with the inverse trend observed in our primary analysis (39). VVC may involve an antifungal mucosal immune response, including IL-17/Th17-related pathways (40). This immune milieu may be less favorable for hrHPV establishment.

Few studies have evaluated HPV infection according to mixed vaginitis combinations. A hospital-based study identified BV + AV as the predominant mixed-vaginitis subtype, but did not find significant differences in HPV infection rates among mixed-vaginitis subgroups (10). In our study, BV + VVC was the most frequent mixed subtype, and hrHPV positivity differed significantly across mixed vaginitis groups. Microbiome studies have also reported higher levels of bacteria commonly enriched in vaginal dysbiosis, such as *Gardnerella* and *Prevotella*, in HPV-positive women (41). These findings are compatible with the possibility that a complex vaginal microbial environment may be involved in hrHPV detection.

Our results indicate that vaginal microecological abnormalities may have different relationships with hrHPV detection across vaginitis subtypes and hrHPV genotype categories. In particular, the observed differences between HPV16/18 and hrHPV12 highlight the need to consider hrHPV genotype category when interpreting the relationship between vaginal microecological status and hrHPV detection.

This study has several strengths. First, using a large Chinese clinical sample, the present study characterized the age-specific distribution of several common types of vaginitis. Second, both vaginal microecological assessment and hrHPV testing were performed during the same hospital visit as part of routine clinical care, which enhanced the comparability of the measurements. Finally, the dataset allowed us to evaluate the associations between hrHPV infection and multiple vaginal microecological conditions, including BV, AV, TV, VVC, and mixed infections.

Our study has several limitations. This was a retrospective and cross-sectional study based on routine clinical and laboratory records. Because vaginal and cervical specimens were both collected during the same hospital visit, this study could not determine the temporal sequence or causal relationship between vaginal microecological abnormalities and hrHPV infection, nor could it determine whether both were driven by shared behavioral, reproductive, or host-related factors. Although the multivariable models adjusted for variables, several established or potential confounders of hrHPV infection were not available, including sexual behavior, number of sexual partners, condom use, smoking, parity, contraceptive use, menopausal status, socioeconomic factors, and HPV vaccination status. Residual and unmeasured confounding therefore cannot be excluded, and the adjusted associations should not be interpreted as causal or fully independent effects.

Because participants were recruited from hospital-based gynecologic, reproductive medicine, and health screening services with mixed clinical indications, selection bias may have occurred. The findings may not be directly generalizable to the broader female population. In addition, the exclusion criteria were ascertained retrospectively from available clinical records, and incomplete documentation of recent sexual activity, vaginal douching, medication use, or other clinical factors may have led to misclassification.

The age groups were imbalanced in size, especially the ≤20-year subgroup, and several vaginitis subgroups had small counts, including TV, BV + TV, AV + TV, and BV + AV + TV. These sparse categories may have reduced statistical power and limited the precision and stability of subgroup estimates. Cervical cytology, histopathological outcomes, and longitudinal type-specific HPV follow-up data were not available, so we could not evaluate hrHPV persistence, clearance, or cervical lesion progression. Finally, because multiple subgroup and genotype-category comparisons were performed without formal correction for multiple testing, some statistically significant findings may represent chance findings.

Taken together, this retrospective study based on same-visit vaginal and cervical sampling identified age-related variation in both hrHPV positivity and vaginal microecological abnormalities among women from Eastern China. A distinctive contribution of this study is that, using a large hospital-based clinical laboratory population, we simultaneously analyzed common vaginitis subtypes, mixed-vaginitis patterns, and clinically relevant hrHPV genotype categories. Women aged ≤20 years had the highest hrHPV positivity, together with relatively high detection rates of BV, TV, and VVC, supporting the continued relevance of early HPV vaccination and targeted sexual health education in young women. The modest increase in hrHPV positivity and the relatively high proportion of HPV16/18 among older women also indicate that HPV-based screening should not be overlooked in older age groups. The associations between vaginal microecological status and hrHPV positivity differed by vaginitis subtype and hrHPV genotype category, suggesting that age, genotype category, and vaginal microecological status may provide useful contextual information when interpreting hrHPV screening results and managing HPV-positive women, particularly those with BV, TV, or inflammatory indicators. However, these findings do not directly support changes to existing HPV screening protocols, nor do they establish that prevention or correction of vaginal dysbiosis can reduce hrHPV acquisition, promote clearance, or reduce cervical lesion progression. Future longitudinal cohort and interventional studies incorporating vaccination history, detailed behavioral and reproductive factors, extended HPV genotyping, repeated vaginal microecological assessment, and cervical lesion outcomes are needed to clarify the temporal sequence and clinical significance of these associations.

## Data Availability

The original contributions presented in the study are included in the article/Supplementary material, further inquiries can be directed to the corresponding author.
